# Complementary DNA Significantly Enhancing Signal Response and Sensitivity of a Molecular Beacon Probe to Aflatoxin B1

**DOI:** 10.3390/bios13020195

**Published:** 2023-01-28

**Authors:** Chao Wang, Kexiao Zhu, Jie Yu, Pengfei Shi

**Affiliations:** 1 College of Medicine, Linyi University, Linyi 276005, China; 2 College of Chemistry and Chemical Engineering, Linyi University, Linyi 276005, China

**Keywords:** aflatoxin B1, aptamer, molecular beacon, complementary DNA, enhanced signal

## Abstract

This paper reported an improved molecular beacon method for the rapid detection of aflatoxin B1 (AFB1), a natural mycotoxin with severe carcinogenicity. With the assistance of a complementary DNA (cDNA) chain, the molecular beacon which consists of a DNA aptamer flanked by FAM and BHQ1 displayed a larger fluorescent response to AFB1, contributing to the sensitive detection of AFB1. Upon optimization of some key experimental factors, rapid detection of AFB1 ranging from 1 nM to 3 μM, within 20 min, was realized by using this method. A limit of detection (LoD) of 1 nM was obtained, which was lower than the LoD (8 nM) obtained without cDNA assistance. This aptamer-based molecular beacon detection method showed advantages in easy operation, rapid analysis and larger signal response. Good specificity and anti-interference ability were demonstrated. This method showed potential in real-sample analysis.

## 1. Introduction

A simple, rapid and on-site detection method protocol is more desired for the sensing of toxins and hazardous chemicals. Aflatoxin B1 (AFB1) is a natural mycotoxin commonly found in fungus colonized cereals (e.g., corn, barley, wheat, peanut, bean, etc.) and their products [1]. The International Agency for Research on Cancer (IARC) has classified AFB1 as a Group 1 carcinogen due to its definite carcinogenesis [2,3]. AFB1 monitoring is an effective way to prevent contaminated samples from entering the human food chain and to reduce AFB1 exposure [4,5]. To date, however, routine detection methods for AFB1 including HPLC and LC-MS have been limited to complex sample pretreatment, sophisticated instruments and professional staff [6,7], resulting in these methods being unserviceable in some practical on-site and rapid analysis cases. The antibody-based immunoassay technique provides an alternative, which allows for the simple, rapid and in situ detection of AFB1 [8,9]. However, the preparation of antibodies is time-consuming and costly, and antibodies are vulnerable to denaturing. It is more difficult to prepare antibodies specific to toxic matter than non-toxic substances. These shortcomings restrict the wide application of immunoassays in toxins and hazardous chemical sensing. Therefore, it is desirable to develop new AFB1 detection methods with characteristics of easy operation and rapid and low-cost analysis.

Aptamer is an oligonucleotide that holds high specificity and binding affinity toward a wide range of targets, which is isolated from a combinatorial DNA library via a selection technology named systemic evolution of ligands by exponential enrichment (SELEX) [10,11,12]. Aptamer provides an ideal alternative of antibody and shows many advantages including chemical synthesis, thermal stability, low cost and easy modification over other antibodies [13,14]. The development of novel aptamer-based detection methods is a future direction for toxins and hazardous chemical sensing [15,16,17,18,19]. In the last decades, many aptamer-based assays/biosensors for AFB1 have been developed [20,21,22]. However, special reagents, repeated incubation and washing are usually needed in these assay/biosensor methods, which might retard their practical use ability in simple and rapid analysis.

The molecular beacon (MB) has been attractive for bioassays for nucleic acids, showing advantages in simplicity, rapidity and sensitivity [23,24,25]. The use of aptamer has allowed one to develop aptamer-based fluorescent molecular beacon assays for the detection of proteins and small molecules [26,27], e.g., Tat protein of HIV-1 [28], thrombin [29,30], cocaine [31], adenosine triphosphate (ATP) [32,33], etc., based on binding-induced structure change and the subsequent fluorescence decrease or fluorescence increase. Previously, we have reported a “signal-off” molecular beacon strategy for the fluorescence detection of AFB1 [34], which has shown advantages in simple operation and rapid analysis. However, we found that there were two facts which would reduce detection performance of this detection strategy: for one, the initial hairpin structure of the molecular beacon (MB) molecule without AFB1 would lead to a low starting fluorescence intensity of MB before adding AFB1; for the other, the intermolecular base-pairing reaction of MB molecules would cause fluorescence quenching before adding AFB1 and invalidate the “signal-off” response of MB to AFB1. Therefore, it is necessary to make some advancements to this detection proposal.

**Scheme 1:**
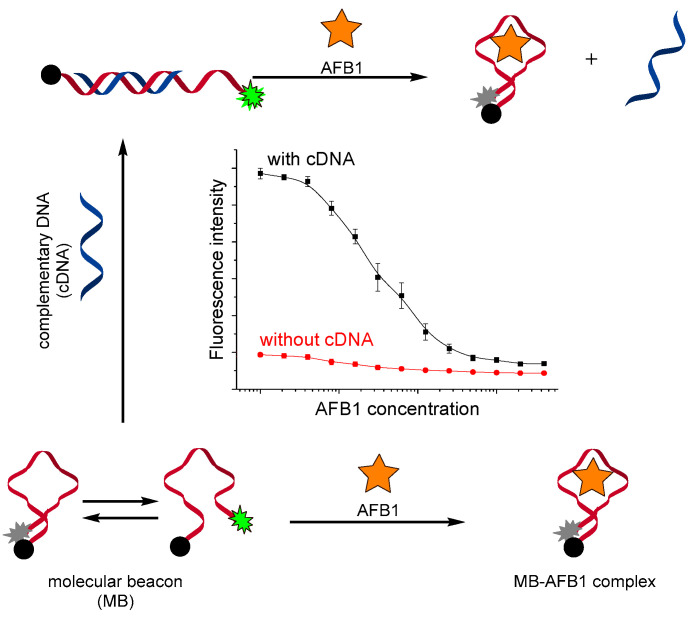
Picture illustrating the principle of this proposal. With the assistance of a complementary DNA (cDNA) strand, the molecular beacon (MB) hybridizes with the cDNA to form a duplex structure, in which FAM labeled at its 3′ end is apart from BHQI labeled at its 5′ end, and there is no fluorescence quenching. Upon the addition of AFB1, the MB prefers to bind with AFB1 rather than cDNA, and adapts into a hairpin structure in which FAM and BHQI are in close proximity, and fluorescence quenching occurs. Detection of AFB1 can be rapidly achieved by measurement of fluorescence intensity decline. Without cDNA, signal response and sensitivity of the MB probe are weak.

Herein, we have presented a complementary DNA (cDNA)-chain-assisted molecular beacon method for the rapid detection of AFB1. As illustrated in Scheme 1, in the absence of AFB1, aptamer hybridized with its complementary DNA (cDNA) strand to form a duplex structure, resulting in fluorescein (FAM) and fluorescent quencher (BHQI), is completely separated, and no fluorescence quenching occurs. Upon the addition of AFB1, aptamer prefers to bind with AFB1 rather than the cDNA strand, forming a stem-loop structure in which FAM and BHQI are in close proximity, and fluorescence quenching occurs. The cDNA strand could reduce fluorescence quenching before the addition of AFB1 and enhance the initial fluorescence strength of the MB probe. With the assistance of a cDNA strand, MBs signal change caused by AFB1 was enhanced and detection performance was improved. Under optimized conditions, we quantitatively achieved the detection of AFB1 in a range of 1 nM to 3 μM, with a detection limit of 1 nM which was lower than that (8 nM) obtained without cDNA’s assistance. The specificity of this method for AFB1 was demonstrated. We successfully detected that AFB1 spiked in 50-fold diluted beer, 50-fold diluted serum and 10-fold diluted tap water by using this method, respectively. This shows its applicable potential in real-sample analysis.

## 2. Materials and Methods

### 2.1. Materials and Reagents

DNA chains listed in Table 1 were synthesized and purified via HPLC by Sangon Bioteh Co., Ltd. (Shanghai, China, https://www.sangon.com/ (accessed on 28 January 2022)). Target mycotoxin aflatoxin B1 (AFB1) and non-target mycotoxins including ochratoxin A (OTA), zearalenone (ZAE) and deoxynivalenol (DON) were obtained from Sangon Bioteh Co., Ltd. (Shanghai, China, https://www.sangon.com/ (accessed on 28 January 2022)). Normal human serum was purchased from Beijing Solarbio Life Science Co., Ltd. (Beijing, China, https://www.solarbio.com/ (accessed on 28 January 2022)). Beer was bought from a local supermarket. Assay buffer (pH 7.5) contained 20 mM Tris-HCl, 200 mM NaCl and 20 mM MgCl_2,_ and 0.01% (*v*/*v*) tween20 was used for analysis. Aqueous solutions were prepared with ultrapure water where resistance was more than 18.2 MΩ·cm.

### 2.2. Analysis Procedures for AFB1 Detection

Firstly, the AFB1 sample, MB probe and cDNA chain were mixed in assay buffer solution. Unless otherwise specified, final concentrations of MB probe and cDNA were 20 nM. After incubation for 20 min at 4 °C, 200 μL of the reaction mixture solution was transferred into a microvolume quartz cuvette with 10 mm pathway length, and fluorescence intensity was measured immediately using a fluorescence spectrophotometer (Edinburgh FLS980-STM, U.K.). For fluorescence intensity measurement, the excitation wavelength was 485 nm and the emission wavelength was 518 nm. Three repeated measurements were carried out and the average data were used for quantitative analysis of AFB1.

### 2.3. Specificity Tests

To assess specificity of this proposal method, non-target mycotoxins including OTA, ZAE, DON and their mixtures were tested as interferences. In detail, these non-target mycotoxins were mixed with MB and cDNA. After 20 min incubation at 4 °C, the fluorescence intensity of MB was measured. The analysis procedures and conditions were the same as described above for the AFB1 detection.

### 2.4. Detection of AFB1 Spiked in Complex Matrix

We detected different concentrations of AFB1 spiked in 50-fold diluted beer, 50-fold diluted human serum and 10-fold diluted tap water, respectively. Prior to analysis, the beer, serum and tap water were ultrasonicated to degas, and then filtered through a syringe filter (0.22 μm) before dilution with the assay buffer. After dilution via the assay buffer and adulteration with standard AFB1 solution, the filtrate was used as a complex matrix sample for analysis.

## 3. Results and Discussions

### 3.1. Inspiration and Feasibility of This Proposal

**Figure 1:**
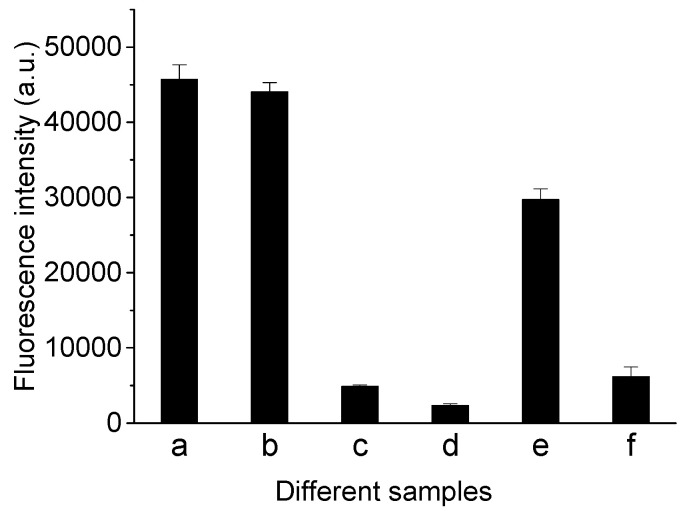
Fluorescence intensities of samples containing different reagents: (**a**) FDNA; (**b**) FDNA and BDNA; (**c**) MB; (**d**) MB and AFB1; (**e**) MB and cDNA and (**f**) MB, cDNA and AFB1. Concentrations of FDNA, BDNA and MB were 20 nM. Concentration of AFB1 was 500 nM. Excitation and emission wavelengths set for fluorescence measurements were 485 nm and 518 nm.

According to the previous reports, the anti-AFB1 aptamer selected by Le et al. has a hairpin structure that is critical to its binding function; we have fabricated a molecular beacon (MB) capable of rapid analysis of AFB1, using a deliberately designed aptamer with a hairpin structure. This MB could bind with the AFB1 molecule through chemical forces including hydrogen bond, π-π stacking, electrostatic, van der Waals and hydrophobic interactions, and an MB conformational change occurred. As an assumption (bottom section of Scheme 1), the presence of AFB1 would cause the MB molecule to adjust into a stable stem-loop structure, in which FAM would be close to BHQ1 and fluorescence quenching would occur so that AFB1 could be detected by measuring the reduction in fluorescence intensity. However, we found that a large proportion (~90%) of FAM fluorescence had been quenched before the addition of AFB1, causing poor efficiency. This may have resulted from two possibilities: (a) most of the MB molecules stayed in a tertiary structure which brought FAM and BHQ1 to be adjacent in space, rather than in a formation that separated FAM and BHQ1, in the absence of AFB1; (b) intermolecular hybridization occurred among MBs, which caused FAM from one MB molecule and BHQ1 from another MB molecule to come into a close, causing fluorescence quenching. To verify or falsify these possibilities, we measured the fluorescence intensities of samples containing different reagents (Figure 1). The fluorescence intensity of the sample containing only MB (Figure 1c) is much lower than that of the sample containing only FDNA (Figure 1a), which is similar to that of the sample containing FDNA and BDNA (Figure 1b). These results imply that MB molecules had already stayed in a hairpin structure before the addition of AFB1, which is the major reason that caused low initial fluorescence intensity. This major reason further resulted in less of a reduction in fluorescence intensity upon the addition of AFB1. To resolve the problem and enhance the fluorescence response, we employed a cDNA chain to hybridize with MB and hoped to increase the initial fluorescence intensity before the addition of AFB1. As a result, fluorescence intensity of the sample containing only MB and cDNA (Figure 1e) was obviously higher than that of the sample containing only MB (Figure 1c), meaning this resolution is viable. Then, AFB1 was added. The fluorescence intensity of the sample containing MB, cDNA and AFB1 (Figure 1f) decreased in comparison to that of the sample containing MB and cDNA (Figure 1e). This means that AFB1 detection could be achieved via this proposal. 

### 3.2. Optimization of Experimental Conditions

**Figure 2:**
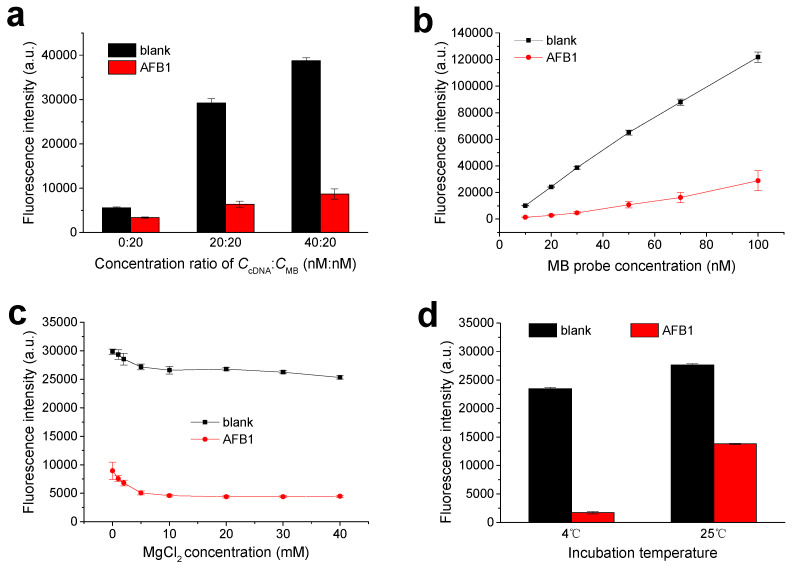
Optimizations of experimental conditions. (**a**) Fluorescence intensity of samples with or without AFB1 obtained with different concentration ratios between cDNA and MB; (**b**) fluorescence intensity of samples with or without AFB1 obtained with different concentrations of MB, when ratio value of *C*_cDNA_:*C*_MB_ was fixed at 1:1; (**c**) fluorescence intensity of samples with or without AFB1 using assay buffer containing different concentrations of MgCl_2_ and (**d**) fluorescence intensity of samples with or without AFB1 under different incubation temperatures.

We optimized some key factors of this experiment. Figure 2a depicts the influences of the concentration ratio between cDNA and MB (*C*_cDNA_:*C*_MB_) on the detection performance of this proposal. The fluorescence intensity of the sample without AFB1 (F_blank_) and the fluorescence intensity of the sample containing AFB1 (F_AFB1_) both increased by increasing the ratio of *C*_cDNA_:*C*_MB_, demonstrating that much more MB hybridized with cDNA. However, too much cDNA might increase the hybridization reaction possibility of MB and subsequently decrease its binding with AFB1. To obtain a better competition between cDNA and AFB1 for MB, the optimal *C*_cDNA_:*C*_MB_ was determined to be 20 nM:20 nM, as the signal descent degree (1 − (F_AFB1_/F_blank_)) × 100% caused by AFB1 approached the maximum at this ratio (Figure S1 in the electronic Supplementary Material (ESM)).

The influences of molecular beacon (MB) probe concentration on detection performance were researched under the *C*_cDNA_:*C*_MB_ ratio value fixed at 1:1. The results are shown in Figure 2b. F_blank_, F_AFB1_ and fluorescence reduction (F_blank_-F_AFB1_) caused by AFB1 all increased with an increase in MB concentration. Finally, 20 nM MB was chosen to be used, since signal descent degree (1 − (F_AFB1_/F_blank_)) × 100% was largest at this concentration (Figure S2 in ESM).

Effects of cations (Mg^2+^ and Na^+^) in assay buffer were also tested. Figure 2c shows the effects of Mg^2+^ cations in assay buffer on detection performance. In a lower MgCl_2_ concentration range, F_blank_ and F_AFB1_ all decreased with an increasing concentration of MgCl_2_. This might be due to the fact that Mg^2+^ can induce MB to form a stable hairpin structure, which causes fluorescence quenching. When 20 mM MgCl_2_ was used, the value of (1 − (F_AFB1_/F_blank_)) × 100% was the largest (Figure S3 in ESM), meaning better sensitivity. Therefore, assay buffer containing 20 mM MgCl_2_ was used in this study. Figure S4 in ESM shows the effects of NaCl in assay buffer containing 20 mM MgCl_2_. When AFB1 was absent, a proper amount of NaCl could reduce the non-specific adsorption. Finally, NaCl at 200 mM in the assay buffer was chosen and applied to the further experiments.

The experimental temperature, which could transform DNA conformation and subsequently alter the binding affinity of the aptamer target, was also optimized. A certain concentration of AFB1 was detected under different incubation temperatures of 4 °C and 25 °C, respectively (Figure 2d). A larger signal change (F_blank_-F_AFB1_) was obtained at 4 °C, meaning better sensitivity. 

### 3.3. Sensitivity Analysis of Novel Aptameric Sensor against AFB1

**Figure 3:**
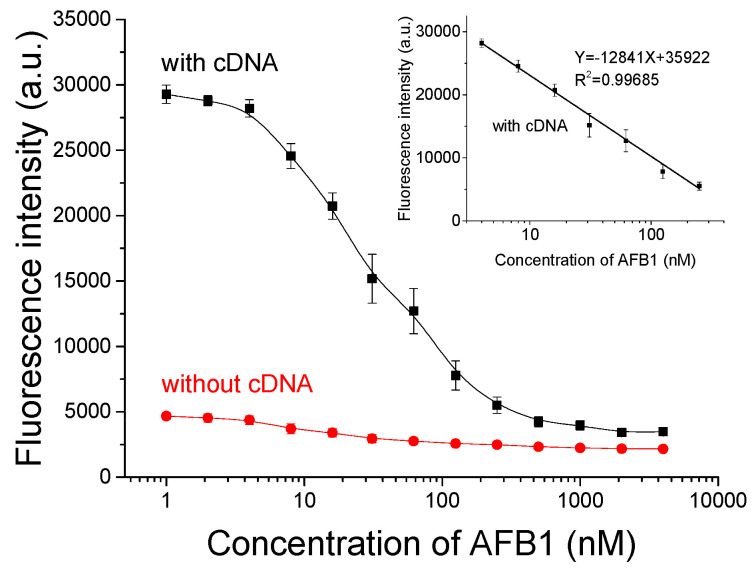
Fluorescence intensities corresponding to different concentrations of AFB1, with or without cDNA assistance. Assay buffer (pH 7.5) containing 20 mM Tris-HCl, 20 mM MgCl_2_, 200 mM NaCl and 0.1% (*v*/*v*) tween20 was used. MB probe concentration was 20 nM. cDNA concentration was 20 nM. Excitation/emission was 485 nm/518 nm. Incubation was carried out at 4 °C for 20 min.

Under optimal conditions obtained above, we detected different concentrations of AFB1 using this MB with or without cDNA, respectively. As Figure 3 shows, with the assistance of the cDNA chain, the MB generated a much higher initial fluorescence intensity before the addition of AFB1, in comparison to that without cDNA assistance. Additionally, a more-than-ten-times-larger decrease in fluorescence intensity caused by the addition of AFB1 was observed when cDNA was used, which means an enhanced response and better sensitivity. In the concentration range of 1 nM to 125 nM, a linear relationship between fluorescence intensity and AFB1 concentration (Y = 12841X + 35922, R^2^ = 0.99685, where Y is the fluorescence intensity and X is the logarithm of AFB1 concentration) was obtained with cDNA assistance. The limit of detection (LOD) determined by signal/noise being more than three (S/N > 3) was 1 nM, lower than the LOD (8 nM) obtained without cDNA assistance. This detection performance is better than or comparable to some of the previous literature listed in Table S1. Additionally, this aptasensor shows advantages in easy operation, rapid analysis and large signal change.

### 3.4. Specificity of This Detection Method

**Figure 4:**
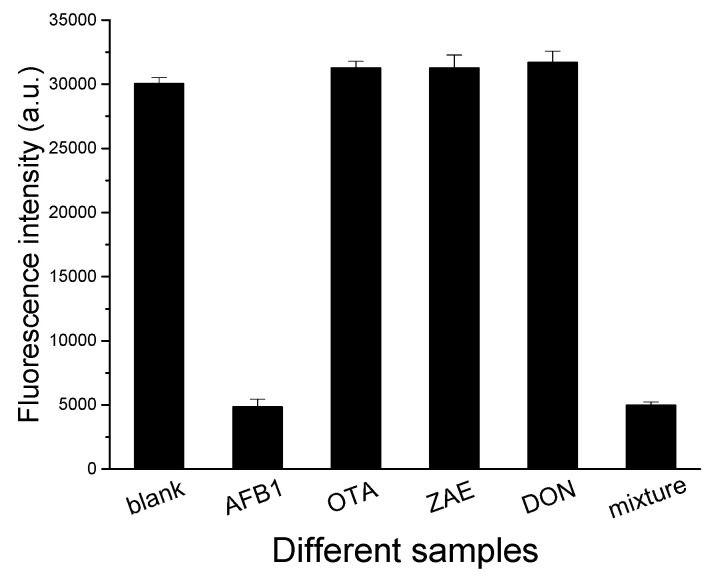
Fluorescence intensities corresponding to samples containing different mycotoxins. Assay buffer (pH 7.5) containing 20 mM Tris-HCl, 20 mM MgCl_2_, 200 mM NaCl and 0.1% (*v*/*v*) tween20 was used. Concentrations of MB probe and cDNA were 20 nM. AFB1 concentration was 500 nM. Concentrations of OTA, ZAE and DON were 1 μM. Excitation/emission was 485 nm/518 nm. Incubation was carried out at 4 °C for 20 min.

To demonstrate the specificity of this method for AFB1 detection, some non-target mycotoxins were also detected using this method. Results are shown in Figure 4. In comparison with the blank sample, AFB1 addition caused an obvious decrease in the fluorescence intensity. In contrast, the addition of these non-target mycotoxins OTA, ZAE and DON caused a neglectful change in fluorescence intensity, compared with the blank sample. Co-exists of these non-target mycotoxins had no interference on AFB1 detection. These results imply a good specificity of this method toward AFB1.

### 3.5. Complex Matrix Interference Tests

**Figure 5:**
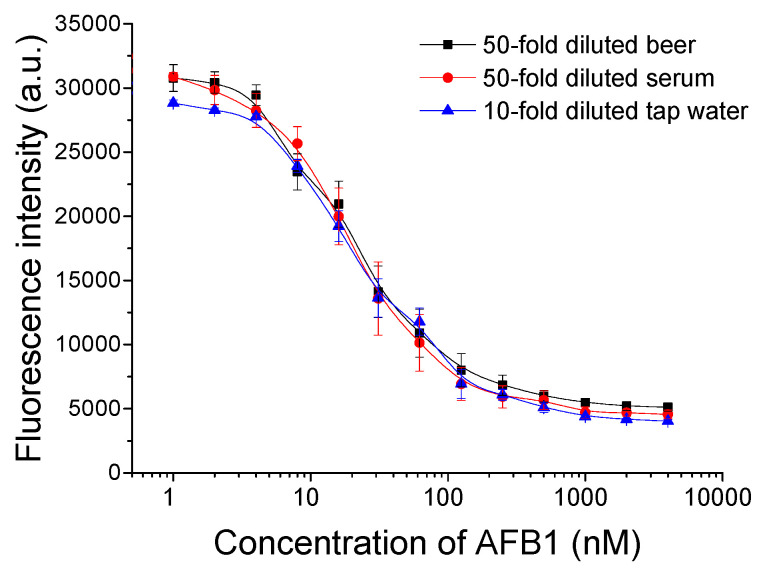
Fluorescence intensities corresponding to various concentrations of AFB1 spiked in 50-fold diluted beer, 50-fold diluted serum and 10-fold diluted tap water, respectively.

To assess the application ability of this detection protocol in a complex matrix, different concentrations of AFB1 spiked in 50-fold diluted beer, 50-fold diluted serum and 10-fold diluted tap water were detected by using this method, respectively (Figure 5). Corresponding to any of these matrixes, fluorescence intensity declined with the increasing spiked amount of AFB1. A dynamic range of 1 nM to 3 μM and LOD of 1 nM were also achieved. These detection performances were comparable to those in a pure assay buffer system. These results imply the good anti-interference ability of this method, and its application potential in real-sample analysis.

## 4. Conclusions

We have developed a simple aptamer-based molecular beacon method for the rapid detection of AFB1, in which a cDNA chain was employed to increase the initial fluorescence intensity of a molecular beacon (MB) probe before the addition of AFB1. Compared with the use of the MB probe alone, a larger fluorescence signal change caused by AFB1 and a lower detection limit were obtained, with the assistance of a cDNA chain. The detection range of this proposed method was 1 nM to 3 μM AFB1. This method showed good specificity toward AFB1, and resistance ability to the complex matrix interference.

## Figures and Tables

**Table 1:** Information of different DNA chains.

Name	Base Sequence (5′ to 3′)	Labels
MB	CGTGTTGTCTCTCTGTGT CTCG	5′-BHQ1, 3′-FAM
FDNA	CGTGTTGTCTCTCTGTGT CTCG	3′-FAM
BDNA	CGTGTTGTCTCTCTGTGT CTCG	5′-BHQ1
cDNA	GAGACAACACG	no

BHQ1 is the abbreviation for black hole quencher Ⅰ; FAM is the abbreviation for fluorescein.

## Supplementary Materials

The following supporting information can be downloaded at https://www.mdpi.com/article/10.3390/bios13020195/s1: Figure S1: selection of concentration ratio between molecular beacon and complementary DNA strand; Figure S2: selection of MB concentration; Figure S3: optimization of MgCl2 concentration in assay buffer; Figure S4: effects of NaCl concentration in assay buffer on detection performance of this proposal [35,36,37,38,39,40,41].
