# Supplementary material for: Complementary DNA Significantly Enhancing Signal Response and Sensitivity of a Molecular Beacon Probe to Aflatoxin B1
Source: Biosensors (Basel). 2023 Jan 28;13(2):195. doi: 10.3390/bios13020195 (PMC9953557; doi:10.3390/bios13020195)
Supplement: Supplementary file 1 [file biosensors-13-00195-s001.zip › biosensors-2116089-supplementary.pdf]

Electronic Supplementary Material

Complementary DNA significantly enhanced signal response and sensitivity of a molecular beacon probe to aflatoxin B1

Chao Wang [1], Kexiao Zhu [2], Jie Yu [2] and Pengfei Shi [1,*]

1 College of Medicine, Linyi University, Linyi 276005, China

2 College of Chemistry and Chemical Engineering, Linyi University, Linyi 276005, China

* Correspondence: shipengfei913@163.com;

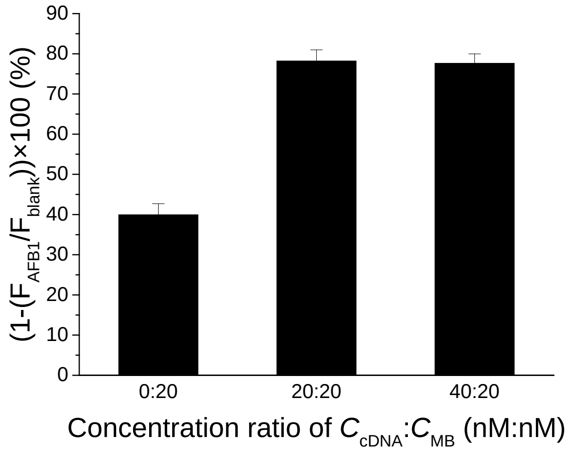

Fig. S1. Selection of concentration ratio between molecular beacon and complementary DNA strand. Assay buffer (pH 7.5) containing 20 mM Tris-HCl, 20 mM $MgCl_2$, 200 mM NaCl and 0.1% (v/v) tween20 was used. AFB1 concentration was 500 nM. Excitation/emission was 485 nm/518 nm. Incubation at 4 ^{o}C was applied.

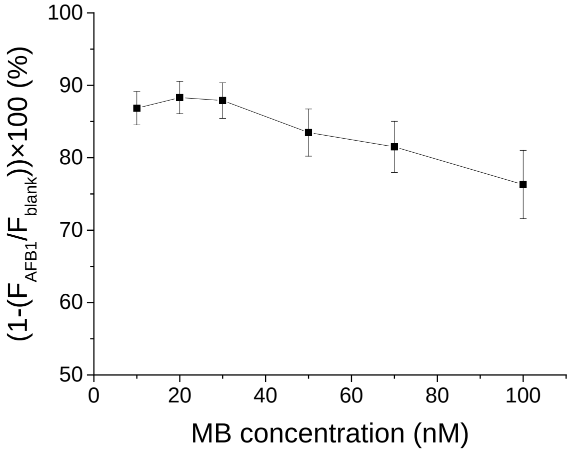

Fig. S2. Selection of MB concentration. Assay buffer (pH 7.5) containing 20 mM Tris-HCl, 20 mM $MgCl_2$, 200 mM NaCl and 0.1% (v/v) tween20 was used. MB probe concentration was 20 nM. cDNA concentration was 20 nM, AFB1 concentration was 500 nM. Excitation/emission was 485 nm/518 nm. Incubation at 4 ^{o}C was applied.

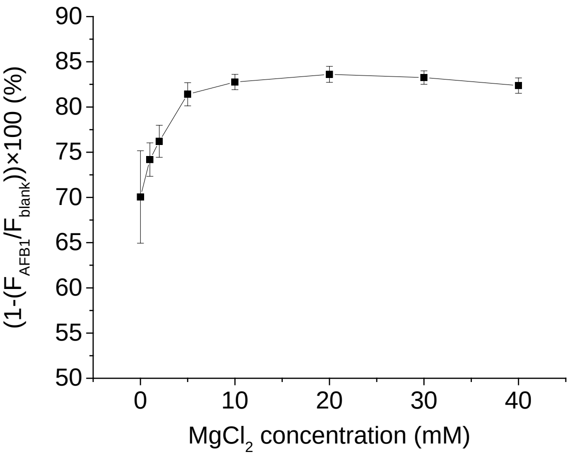

Fig. S3. Optimization of $MgCl_2$ concentration in assay buffer. Assay buffer (pH 7.5) containing 20 mM Tris-HCl, 200 mM NaCl, 0.1% (v/v) tween20 and different $MgCl_2$ concentrations was used. MB probe concentration was 20 nM, cDNA concentration was 20 nM, AFB1 concentration was 500 nM. Excitation/emission was 485 nm/518 nm. Incubation at 4 ^{o}C was applied.

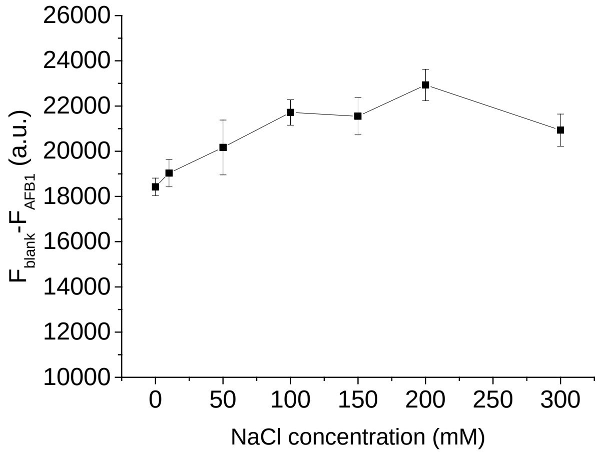

Fig. S4. Effects of NaCl concentration in assay buffer on detection performance. Assay buffer (pH 7.5) containing 20 mM Tris-HCl, 20 mM $MgCl_2$, 0.1% (v/v) tween20 and different NaCl concentrations was used. MB probe concentration was 20 nM. cDNA concentration was 20 nM, AFB1 concentration was 500 nM. Excitation/emission was 485 nm/518 nm. Incubation at 4 ^{o}C was applied.

Table S1 Comparison of some aptamer-based sensors for AFB1

Strategies	Detection range	LOD	References
Assay using fluorescein-labeled aptamer and quencher labeled complementary DNA (cDNA)	16-320 nM	5.1 nM	[1]
Fluorescent aptasensor using AIEgens and graphene oxide	0.8-10 nM	0.8 nM	[2]
FRET-based aptasensor	10-400 nM	3.4 nM	[3]
Aptamer with dual-terminal proximity structures	3.2-3200 nM	2.9 nM	[4]
Assay using a quencher system composed of quantum dots and graphene oxide	3.2 nM-320 μM	1.0 nM	[5]
Aptamer assay using aggregation-induced emission dyes	1.0 nM-16 μM	1.0 nM	[6]
An aptamer-based lateral flow strip	--	0.3 nM	[7]
A simple molecular beacon probe enhanced with cDNA	1 nM-3 μM	1 nM	This work

References

1. Chen, L.; Wen, F.; Li, M.; Guo, X.; Li, S.; Zheng, N.; Wang, J., A simple aptamer-based fluorescent assay for the detection of aflatoxin B1 in infant rice cereal. *Food chemistry* **2017,** 215, 377-382.

2. Jia, Y.; Wu, F.; Liu, P.; Zhou, G.; Yu, B.; Lou, X.; Xia, F., A label-free fluorescent aptasensor for the detection of aflatoxin B1 in food samples using AIEgens and graphene oxide. *Talanta* **2019,** 198, 71-77.

3. Sabet, F. S.; Hosseini, M.; Khabbaz, H.; Dadmehr, M.; Ganjali, M. R., FRET-based aptamer biosensor for selective and sensitive detection of aflatoxin B1 in peanut and rice. *Food chemistry* **2017,** 220, 527-532.

4. Xia, X.; Wang, Y.; Yang, H.; Dong, Y.; Zhang, K.; Lu, Y.; Deng, R.; He, Q., Enzyme-free amplified and ultrafast detection of aflatoxin B1 using dual-terminal proximity aptamer probes. *Food chemistry* **2019,** 283, 32-38.

5. Lu, Z.; Chen, X.; Wang, Y.; Zheng, X.; Li, C. M., Aptamer based fluorescence recovery assay for aflatoxin B1 using a quencher system composed of quantum dots and graphene oxide. *Microchimica Acta* **2014,** 182, 571-578.

6. Zhao, Z.; Yang, H.; Deng, S.; Dong, Y.; Yan, B.; Zhang, K.; Deng, R.; He, Q., Intrinsic conformation response-leveraged aptamer probe based on aggregation-induced emission dyes for aflatoxin B1 detection. *Dyes and Pigments* **2019,** 171, 107767.

7. Mousivand, M.; Javan-Nikkhah, M.; Anfossi, L.; Di Nardo, F.; Salina, M.; Bagherzadeh, K., High performance aptasensing platform development through in silico aptamer engineering for aflatoxin B1 monitoring. *Food Control* **2023,** 145, 109418.
